# Compression Stockings and Pressure Ulcers: Case Series of a Neglected Issue

**DOI:** 10.7759/cureus.1763

**Published:** 2017-10-10

**Authors:** Farooq A Rathore, Faria Ahmad, Omer J Khan

**Affiliations:** 1 Department of Rehabilitation Medicine, PNS Shifa Hospital, DHA II, Karachi 75500, Pakistan; 2 Department of Rehabilitation Medicine, CMH Lahore Medical College and Institute of Dentistry, Lahore, Pakistan; 3 Dept of Rehabilitation Medicine, Armed Forces Institute of Rehabilitation Medicine, Rawalpindi, Punjab, Pakistan

**Keywords:** pressure points, pressure ulcer, bed sore, pressure sore, wound, skin care, critical illness, deep vein thrombosis, complications, compression stocking

## Abstract

Pressure ulcers develop in patients who endure long periods of immobilization, often caused by conditions such as musculoskeletal and neurological diseases. Pressure ulcers adversely affect the patient and increase caregiver burden and healthcare costs. Typical sites for these ulcers include the sacrum, trochanters, and heels; they also occur on the nape of the neck, penis, nostrils, helix of the ear, and upper back. Compression stockings are commonly used to prevent and stop the progression of venous disorders, including deep vein thrombosis, but their role in the development of pressure ulcers is not well known. We describe three case reports of pressure ulcer development due to prolonged application of compression stockings. In each case, the nursing staff who were primarily responsible for the prevention of pressure ulcers applied the stockings continuously without any intermittent relief. Moreover, the stockings did not include manufacturer instructions, such as recommended exposure times and applications. We recommend that nursing staff be trained in pressure relief and prevention of pressure ulcers, including rare occurrences, and that manufacturers give detailed guidance regarding the safe use of compression stockings.

## Introduction

A pressure ulcer (PU) is “a localized injury to the skin and/or underlying tissue usually over a bony prominence as a result of pressure or pressure in combination with shear” [1]. PU occurs in patients who are immobile, unwell, or have a major neurological disease (i.e., spinal injury or stroke). They are associated with morbidity and increased health care costs [2]. Intrinsic risk factors associated with the development of PU include incontinence, smoking, hypo-albuminemia, alcoholism, and diabetes. Extrinsic factors include shear and friction [3]. PUs typically appear over bony prominences like the ischium, sacrum, heel, and trochanter [2]. Atypical pressure ulcers are generally not located over a bony prominence. Instead, they can be found in unusual places, such as the nape of the neck, penis, nostrils, helix of the ears, or the upper back. Nursing staff play a key role in the prevention of PUs. Unfortunately, in most hospitals in Pakistan, this aspect of care is usually neglected because of the high patient-to-nurse ratio, lack of trained nursing staff, and absence of formal PU assessment and monitoring programs. Compression stockings are a “routine” intervention used in many hospitals and extended care facilities to prevent thromboembolism in immobilized patients [4]. Like most routine interventions, however, their use is not always closely monitored. These stockings exert pressure on the limbs and pressure points, which can lead to the development of PUs. We present three case reports that highlight the development of PUs caused by prolonged and continuous application of compression stockings to the lower limbs. The aim of these case studies is to highlight the need for vigilance whilst using compression stockings, including regular inspection once they have been applied. Informed consent was obtained from all patients.

## Case presentation

Case 1

**Figure 1:**
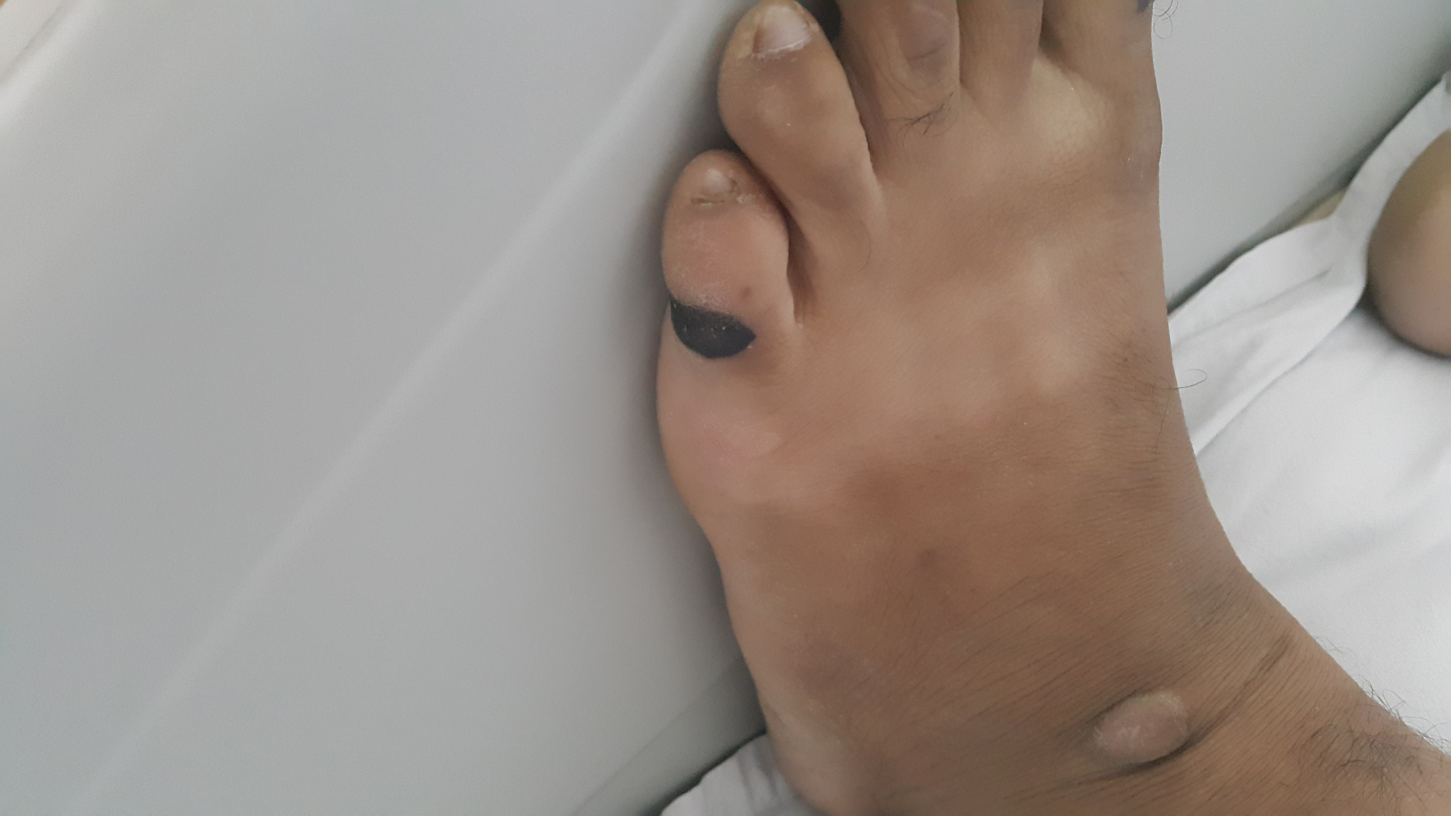
Linear Grade-II pressure ulcers on the lateral aspect of left little toe.

**Figure 2:**
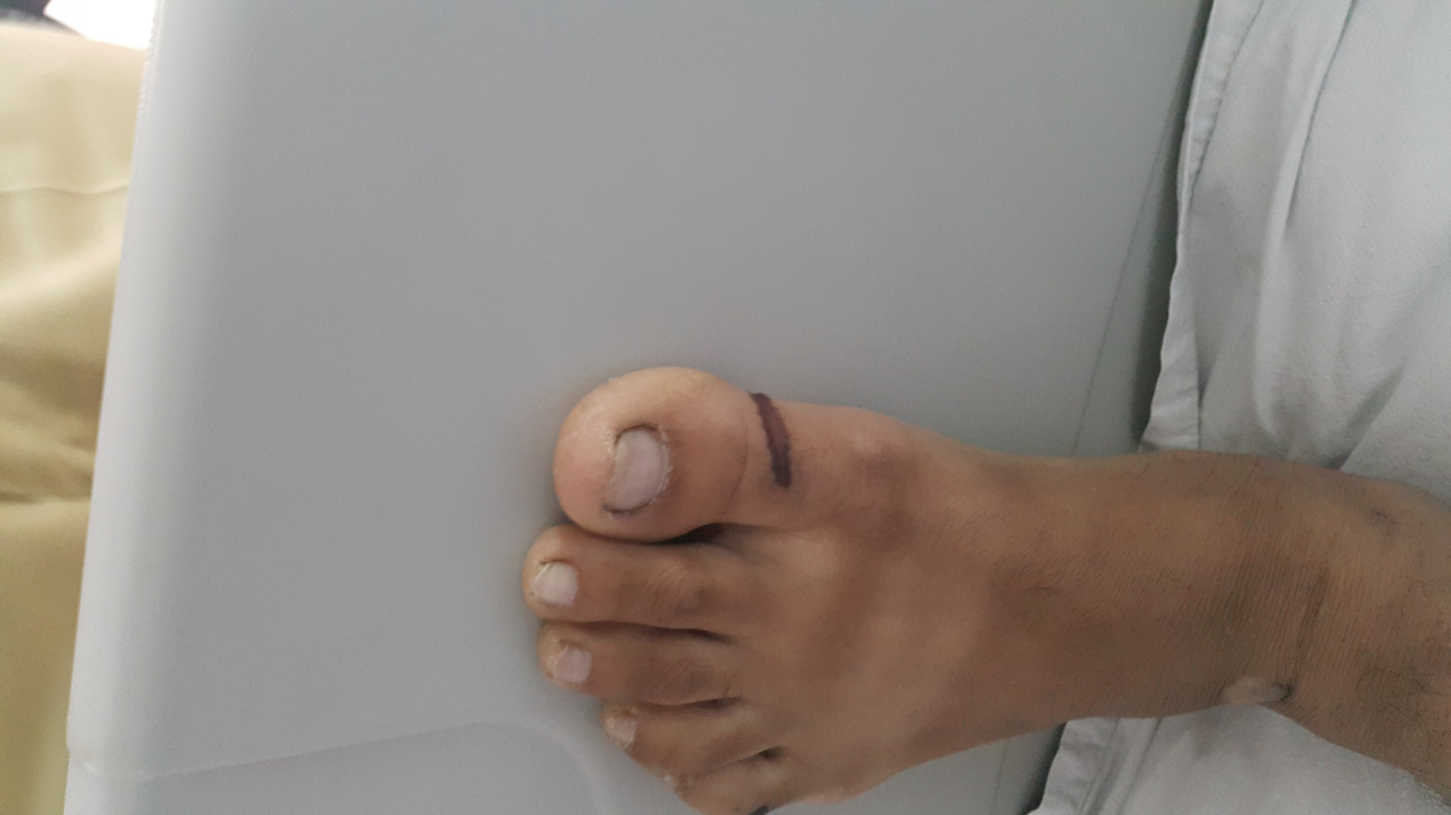
Linear Grade-II pressure ulcers on the medial aspect of left big toe.

**Figure 3:**
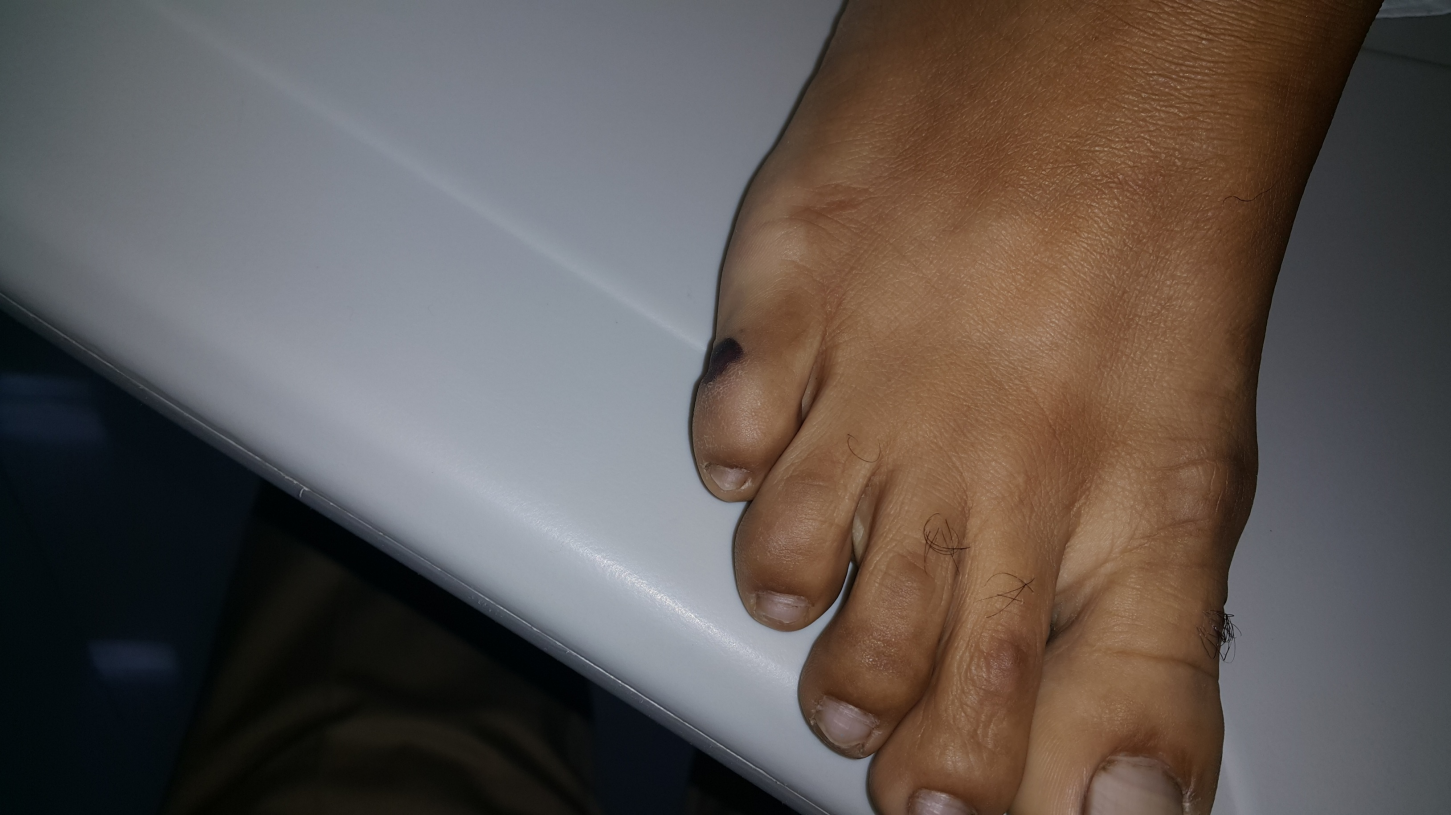
Linear Grade-II pressure ulcers on the lateral aspect of the right little toe.

A 25-year-old, previously healthy male, presented to a peripheral hospital with acute ascending paralysis. There was no history of diarrhea or respiratory infection. He was transferred to our hospital the following day for a neurological consultation and investigation. On arrival, he had dyspnea with the involvement of respiratory muscles. A diagnosis of acute Guillain-Barre syndrome was made, and the patient was placed on a ventilator.The nursing staff applied compression stockings to his lower limbs upon arrival. Removal of the stockings on day 3 of admission revealed linear PUs on the lateral aspect of both little toes and medial aspect of the left and right big toes as seen in Figures 1-3. The ulcers were categorized as Grade II on the National Pressure Ulcer advisory panel scoring system [1].

Upon inquiry, it was revealed by the attendants (family caretaker) that the staff at the previous hospital had applied compression stockings and that they had not been removed nor pressure areas inspected for possible skin break down. Our staff then removed the compression stockings. The nursing staff was advised regarding the need for regular inspection of pressure areas, and the attendants (caretaker and nursing attendant) participated in the pressure ulcer prevention program. The patient’s PUs healed satisfactorily without any intervention.

Case 2

**Figure 4:**
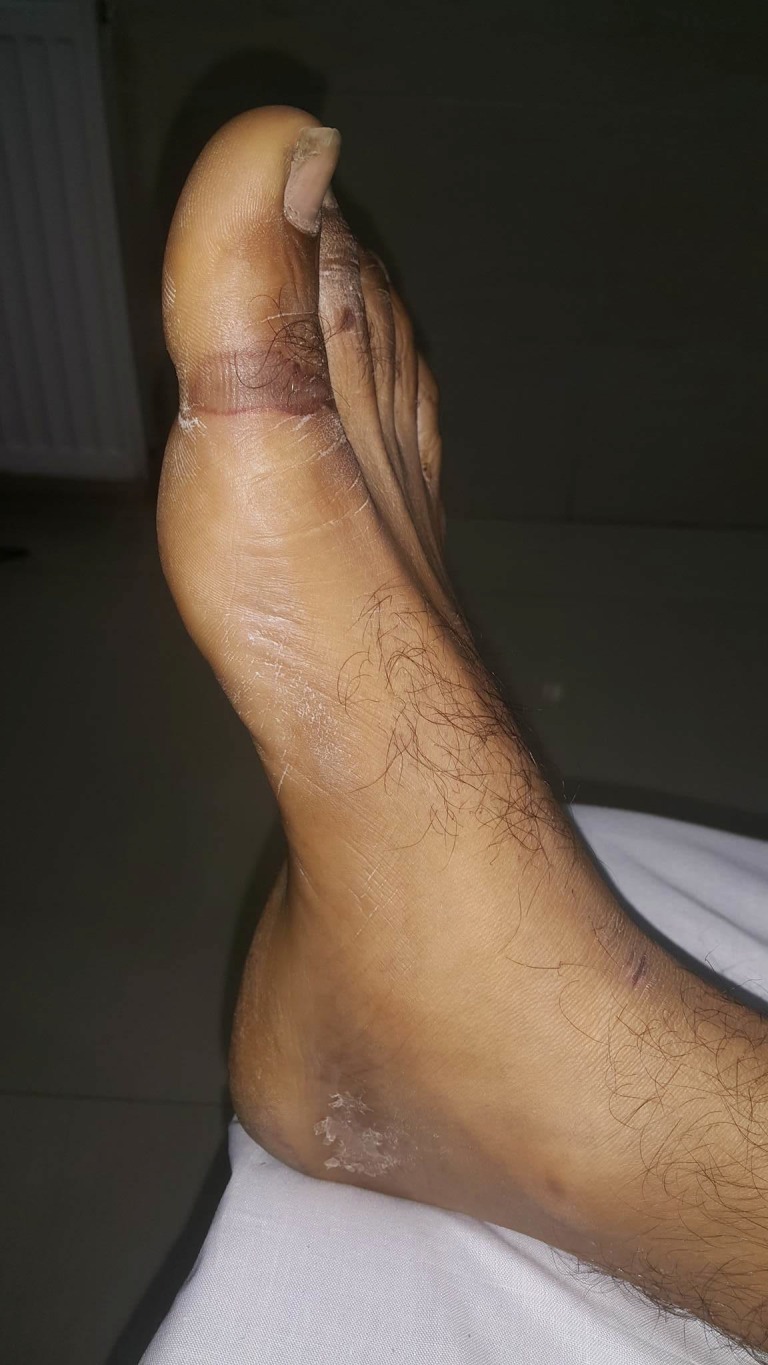
Linear pressure ulcer (Grade-II) on the medial aspect of right big toe.

**Figure 5:**
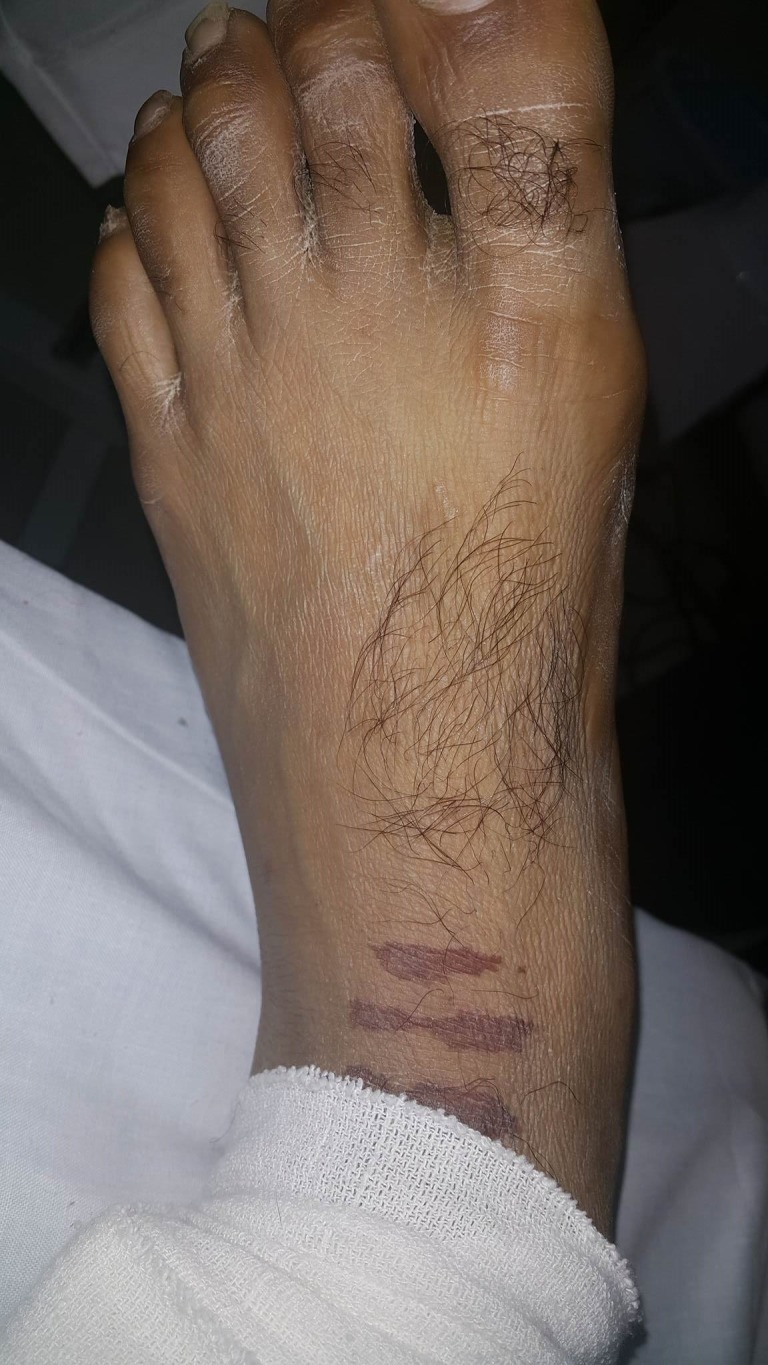
Three pressure ulcers (Grade I) on the anterior aspect of the left ankle.

A 25-year-old male sustained a severe traumatic brain injury and fracture of the shaft of the left femur in a traffic accident. He was transferred to our hospital for orthopedic and neurosurgical consultations following initial treatment at a peripheral hospital. He was placed on ventilator support, and a tracheostomy was performed. He was successfully weaned off the ventilator after 13 days. On his arrival at the intensive care unit, compression stockings were applied to both legs below the knee as mechanical thromboprophylaxis. We noticed a linear, Grade II PU on the medial aspect of the right big toe and three Grade I PUs on the anterior aspect of the left ankle as shown in Figures 4-5.

It was established that prolonged application of the compression stockings caused the ulcers and that no assessment of the feet for possible PU had occurred. Another Grade II PU on the right heel was found, though it was not caused by the application of compression stockings but by poor positioning. All PUs healed without any intervention.

Case 3

**Figure 6:**
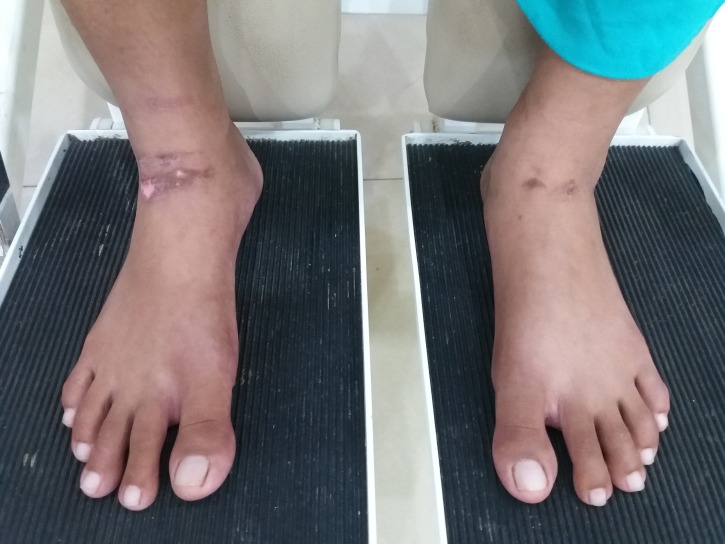
Linear pressure ulcer (Grade-II) on the anterior aspect of both ankles.

A 15-year-old girl was admitted in an unconscious state. She had a high-grade fever and altered sensorium with signs and symptoms suggestive of a neurological dysfunction. Examination and investigations were suggestive of acute encephalomyelitis. She remained hospitalized for three weeks and was sent to our department for rehabilitation and mobility training. On examination, healed Grade II PU on the anterior aspect of both ankles was observed. Upon inquiry, the parents revealed that compression stockings had been applied on the second day of admission and rolled up whenever a physician needed to check the plantar response. The stockings often remained in place for many hours, resulting in PUs at the ankle (Figure 6).

The PUs healed over the next three weeks without any intervention.

## Discussion

These case reports highlight an uncommon complication of a commonly used intervention to prevent thromboembolism. The literature has reported skin breakdown and pressure ulcers due to prolonged application of compression stockings [5]. Yet, most medical and nursing practitioners do not recognize this complication. Apart from a prolonged application, errors in the sizing and application of compression stockings can also contribute to PU development [6]. Moreover, ill-fitting compression stockings can cause common peroneal nerve palsy [7] saphenous nerve injury [8], compartment syndrome [9], and ischemic necrosis of the forefoot [6].

The lack of accuracy in pressure ulcer documentation by nurses has been reported in developing countries as well [10]. In Pakistan, most nurses do not routinely conduct PU risk assessments nor document any PU prevention. Even when a PU is detected, most nurses inform the attending physician but do not actively take part in the management of the PU. Unfortunately, most attending doctors prefer to let the PU heal by itself or to refer the patient to a plastic and reconstructive surgeon. PU documentation is also deficient. Most attending physicians document only the presence of PUs without mentioning important details, such as grade, size, healing status, and condition of surrounding tissue. The adage that “prevention is better than cure” holds true for PU management. Thus, PU management should include monitoring and photography of the ulcers as best practices to reduce inconsistencies between serial assessments and improve the overall care of PUs. Most nurses and doctors in Pakistan do not practice this low-cost solution and important tool for documentation. The only photographic documentation of PUs we witnessed was that of colleagues in plastic and reconstructive surgery.

Preventative care may include spreading awareness via pamphlets and posters in intensive care units. This literature should target nursing staff and attendants (family caregivers). Education about prevention, identification, documentation, and monitoring of PUs also should be included as a mandatory component in nursing training. Moreover, it is imperative for manufacturers of compression stockings to include instructions for regular removal of the stockings to inspect the underlying skin. Such instructions were not included in the manuals for the deep vein thrombosis stockings used on patients in our hospital.

## Conclusions

PUs are preventable. Knowledge and adequate training can increase recognition of early-stage PUs and thus prevent their development, which can markedly reduce their incidence and associated morbidity and mortality risks. PUs can develop at unusual sites; therefore, routine examinations may not suffice in all cases. As demonstrated in the two case studies, compression stockings over pressure points (such as the bony prominences of the toes) can lead to the development of PUs. Constricting equipment, including surgical braces, also can be a risk factor. Thus, nursing and medical staff, along with caregivers, must be vigilant to reduce the risk of this preventable complication.
